# Supplementary material for: Virtual Electrode Recording Tool for EXtracellular potentials (VERTEX): comparing multi-electrode recordings from simulated and biological mammalian cortical tissue
Source: Brain Struct Funct. 2014 May 27;220(4):2333–53. doi: 10.1007/s00429-014-0793-x (PMC4481302; doi:10.1007/s00429-014-0793-x)
Supplement: Supplementary file 1 — Supplementary material 1 (PDF 2973 kb) [file 429_2014_793_MOESM1_ESM.pdf]

ELECTRONIC SUPPLEMENTARY MATERIAL

Virtual Electrode Recording Tool for EXtracellular potentials (VERTEX): Comparing multi-electrode recordings from simulated and biological mammalian cortical tissue

Brain Structure and Function

Richard J Tomsett, Matt Ainsworth, Alexander Thiele, Mehdi Sanayei, Xing Chen, Alwin Gieselmann, Miles A Whittington, Mark O Cunningham* and Marcus Kaiser*

*Joint senior authors

Correspondence:
Dr Marcus Kaiser
School of Computing Science
Newcastle University
Claremont Tower,
Newcastle upon Tyne, NE1 7RU, United Kingdom

Telephone: +44 191 208 8161
Fax: +44 191 208 8232
Email: m.kaiser@ncl.ac.uk

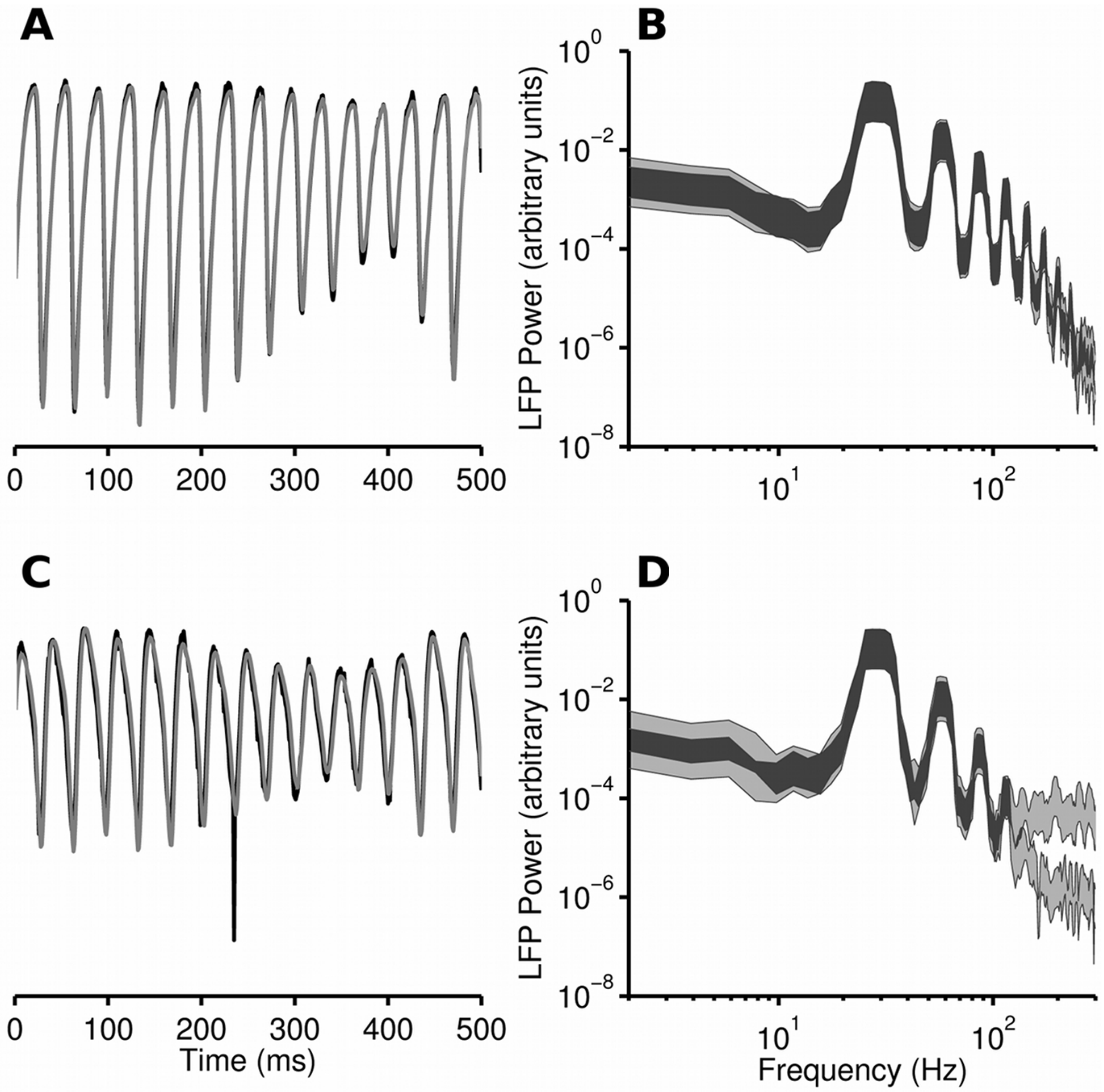

Fig. ESM1 Comparison of simulated LFPs when using purely passive neurons with imported spike times, and when using the AdEx spiking model in the neocortical slice model (related to Results: Spike Import section). Traces in **a** and **c** have been normalised to zero mean, unit standard deviation so that the shape of the LFP signal can be more easily compared, but are otherwise unfiltered. The adaptive current of the AdEx mechanism introduces an offset in the simulated LFPs, but does not dramatically affect the shape of the signal. **a** Simulated LFPs from electrode 42; AdEx version in black, passive version in grey. **b** Power spectral density overlap of these signals (estimated for 500ms signal), with overlapping parts of the estimated spectrum shown in dark grey and non-overlapping shown in light grey. **c** As a, but for electrode 97. Note the small, sharp spike at ~230ms in the AdEx signal. This is a result of the AdEx reset mechanism creating a very short, fast current from a neuron very close to the electrode. **d** As **b**, but for electrode 97. Power spectra diverge above ~100 Hz due to high frequency spike contamination in the AdEx model, but match closely below 100 Hz. We recommend that, while the LFP can be estimated directly when using AdEx spiking networks, results should be checked by using VERTEX's spike import function if a true representation of the synaptic contribution to the LFP is sought at higher frequencies

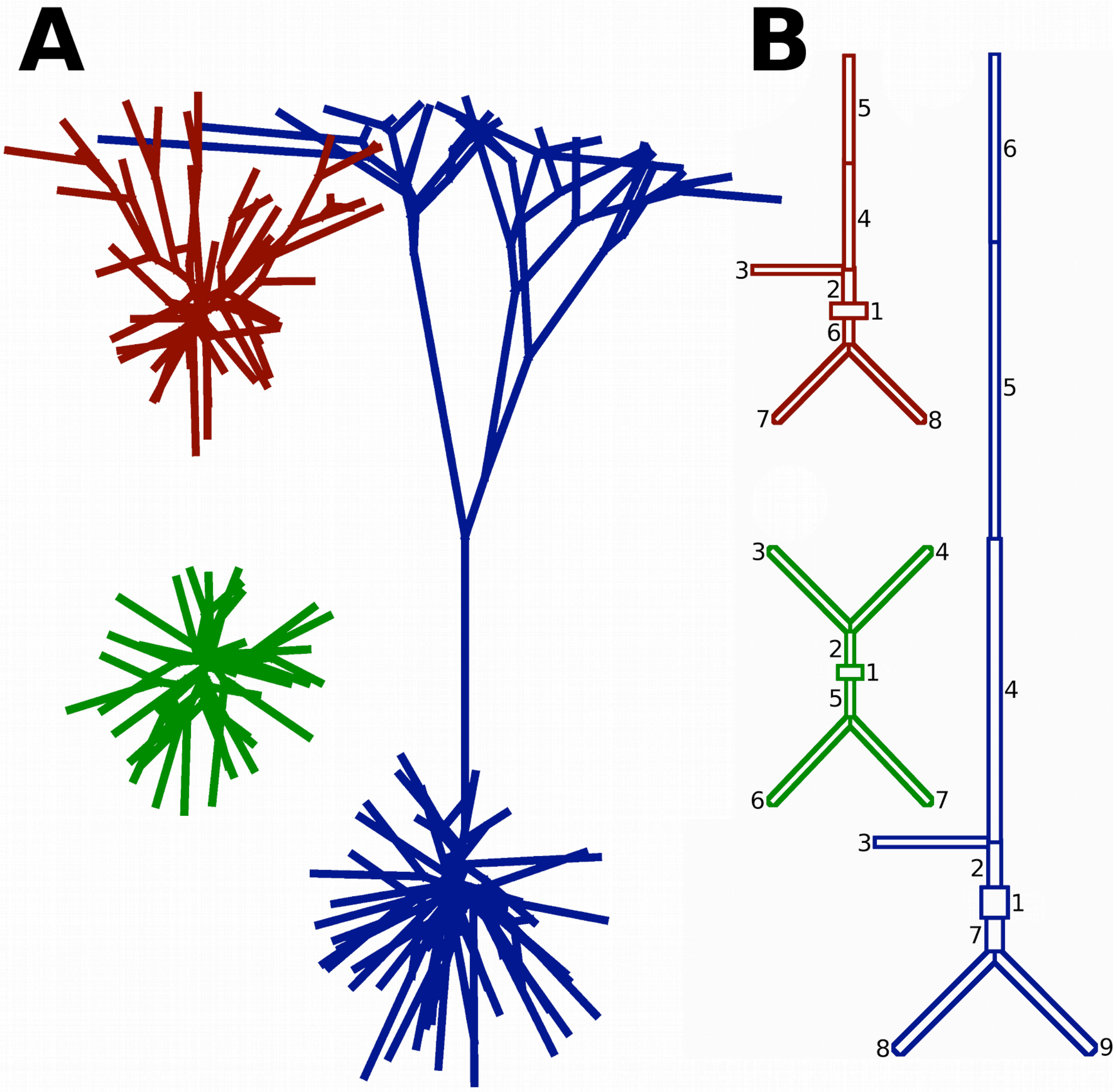

Fig. ESM2 **a** Compartmental structures of morphological cell reconstructions from (Mainen and Sejnowski 1996). The layer 2/3 pyramidal cell, layer 4 spiny stellate cell and layer 5 pyramidal cell are shown in red, green and blue, respectively. **b** Compartmental models reduced from the structures in **a** according to the method in (Bush and Sejnowski, 1993). Compartment numbers correspond to those in Table ESM1

Table ESM1 Reduced neuron model compartment dimensions, used in all reported simulations

	P2/3, P4		P5, P6		SS, B, NB	
Compartment number	Length (μm)	Diameter (μm)	Length (μm)	Diameter (μm)	Length (μm)	Diameter (μm)
1	13	29.80	35	25.00	10	24.00
2	48	3.75	65	4.36	56	1.93
3	124	1.91	152	2.65	151	1.95
4	145	2.81	398	4.10	151	1.95
5	137	2.69	402	2.25	56	1.93
6	40	2.62	252	2.40	151	1.95
7	143	1.69	52	5.94	151	1.95
8	143	1.69	186	3.45	-	-
9	-	-	186	3.45	-	-

Table ESM2 Neuron model parameters, used in all reported simulations (simulations of purely passive neurons only have C_m, R_m, R_a and E_l specified)

**Neuron type**	C_m **(μFcm^{-2})**	R_m **(kΩcm^2)**	R_a **(Ωcm)**	E_l **(mV)**	V_T **(mV)**	Δ_T **(mV)**	α **(nS)**	τ_w **(ms)**	β **(pA)**	v_{reset} **(mV)**
**P2/3, P4**	2.96	6.76	150	-70	-50	2.0	2.60	65	220	-60
**SS4**	2.95	5.12	150	-70	-50	2.2	0.35	150	40	-70
**P5**	2.95	6.78	150	-70	-52	2.0	10.00	75	345	-62
**P6**	2.95	6.78	150	-70	-50	2.0	0.35	160	60	-60
**B**	2.93	5.12	150	-70	-50	2.0	0.04	10	40	-65
**NB**	2.93	5.12	150	-70	-55	2.2	0.04	75	75	-62

Table ESM3 Model composition. Neuron population sizes are given as percentage of total model size. The maximum number of synapses received by a postsynaptic neuron is specified per-layer for pyramidal neurons, whose apical dendrites span several layers. The proportions of these synapses made by each presynaptic neuron group are given in percentages of these maximal synapse numbers. Neurons in the slice model receive fewer than the maximum number of possible synapses because of the effects of slice cutting (see below). Adapted from (Binzegger et al., 2004), with long-range connections removed

Presynaptic neurons

Postsynaptic neurons

		**percent of cells**	**max no. synapses**	**P2/3**	**B2/3**	**nB2/3**	**ss4(L4)**	**ss4(L2/3)**	**p4**	**b4**	**nb4**	**p5(L2/3)**	**p5(L56)**	**b5**	**nb5**	**p6(L4)**	**p6(L56)**	**b6**
**P2/3**	L2/3	26.3	5773	60.1	9.2	4.9	0.6	6.9	7.8	0.8	-	7.5	-	-	-	2.4	-	-
	L1		87	95.1	1.6	-	-	0.3	1.5	0.1	-	1.1	-	-	-	-	-	-
**B2/3**		3.1	3702	53.7	11.0	12.3	0.5	6.1	6.8	0.9	-	6.6	-	-	-	2.1	-	-
**NB2/3**		4.2	3144	57.8	12.6	4.6	0.5	6.6	7.5	0.9	-	7.2	-	-	-	2.2	-	-
**SS4(L4)**		9.3	4113	3.8	0.3	-	16.7	5.2	5.8	12.9	8.1	1.1	0.1	-	-	46.1	-	-
**SS4(L2/3)**		9.3	3610	7.7	0.6	-	15.6	5.3	5.9	12.8	7.6	1.5	0.1	-	-	43.0	-	-
**P4**	L4	9.3	3619	6.0	0.3	-	16.0	5.0	5.8	12.9	8.3	1.6	0.1	0.1	-	43.7	0.1	-
	L2/3		867	63.0	5.1	5.1	0.6	7.2	8.1	0.6	-	7.8	-	-	-	2.5	-	-
	L1		53	6.0	0.3	-	16.0	5.0	5.8	12.9	8.3	1.6	0.1	0.1	-	43.7	0.1	-
**B4**		5.5	2359	8.0	0.7	-	15.1	5.2	5.8	14.8	7.3	1.5	0.1	-	-	41.6	-	-
**NB4**		1.5	2636	3.7	0.3	-	16.3	5.1	5.6	14.8	7.9	1.1	0.1	-	-	45.0	-	-
**P5(L2/3)**	L5	4.9	3971	49.9	2.0	-	3.6	2.2	8.2	1.0	-	12.7	1.1	2.1	12.5	2.5	2.2	-
	L4		198	4.0	0.1	-	17.4	5.4	6.0	9.5	8.4	1.1	0.1	-	-	48.0	-	-
	L2/3		413	62.9	5.1	5.1	0.6	7.2	8.1	0.6	-	7.8	-	-	-	2.4	-	-
	L1		12	97.7	1.7	-	-	0.3	1.5	0.1	-	1.1	-	-	-	-	-	-
**P5(L56)**	L5	1.3	4588	49.3	1.8	-	3.6	2.2	8.1	0.9	-	12.5	1.3	2.0	13.0	2.5	2.8	-
	L4		666	4.0	0.1	-	17.4	5.4	6.0	9.5	8.4	1.1	0.1	-	-	48.0	-	-
	L2/3		1368	63.0	5.1	5.1	0.6	7.2	8.1	0.6	-	7.8	-	-	-	2.5	-	-
	L1		375	95.6	1.7	-	-	0.3	1.5	0.1	-	1.1	-	-	-	-	-	-
**B5**		0.6	2744	49.5	2.5	-	3.6	2.2	8.1	1.2	-	12.6	1.1	2.3	12.3	2.5	2.2	-
**NB5**		0.8	2744	49.5	2.5	-	3.6	2.2	8.1	1.2	-	12.6	1.1	2.3	12.3	2.5	2.2	-
**P6(L4)**	L6	13.8	1326	6.1	0.3	-	1.8	2.1	3.1	0.2	-	0.2	12.0	0.9	-	2.9	32.4	38.1
	L5		979	51.0	0.9	-	3.7	2.3	8.4	0.6	-	13.0	1.1	1.6	12.7	2.5	2.3	-
	L4		1344	4.0	0.1	-	17.4	5.4	6.0	9.5	8.4	1.1	0.1	-	-	48.0	-	-
	L2/3		121	62.9	5.1	5.1	0.6	7.2	8.1	0.6	-	7.8	-	-	-	2.4	-	-
**P6(L56)**	L6	4.6	2264	4.0	0.1	-	17.4	5.4	6.0	9.5	8.4	1.1	0.1	-	-	48.0	-	-
	L5		236	51.0	0.9	-	3.7	2.3	8.4	0.6	-	13.0	1.1	1.6	12.7	2.5	2.3	-
	L4		171	4.0	0.1	-	17.4	5.4	6.0	9.4	8.4	1.1	0.1	-	-	47.8	-	-
	L2/3		286	63.1	5.1	5.1	0.6	7.2	8.1	0.6	-	7.8	-	-	-	2.5	-	-
	L1		4	97.7	1.7	-	-	0.3	1.5	0.1	-	1.1	-	-	-	-	-	-
**B6**		2.0	1310	6.1	0.3	-	1.8	2.1	3.1	0.2	-	0.2	12.0	0.9	-	2.9	32.4	38.1

Table ESM4 Axonal arborisation radii for each neuron group in each layer (mm), adapted from Figure 8 of the supporting information of (Izhikevich and Edelman, 2008). Where no radius was given for a neuron group in a layer in which connections are specified in Table ESM3, we set the radius to 0.05 mm

	L1	L2/3	L4	L5	L6
**P2/3**	0.55	1.12	0.15	1.00	0.15
**B2/3**	0.05	0.50	0.15	0.15	0.05
**NB2/3**	0.20	0.20	0.20	0.20	0.20
**SS4(L4)**	0.05	0.30	1.12	0.40	0.15
**SS4(L2/3)**	0.15	0.40	0.50	0.15	0.15
**P4**	0.15	1.12	0.15	0.55	0.15
**B4**	0.05	0.05	0.50	0.05	0.05
**NB4**	0.20	0.20	0.20	0.20	0.20
**P5(L2/3)**	0.15	0.40	0.30	0.50	0.25
**P5(L5/6)**	0.05	0.05	0.15	0.50	1.00
**B5**	0.05	0.05	0.05	0.5	0.05
**NB5**	0.20	0.20	0.20	0.20	0.20
**P6(L4)**	0.05	0.15	1.00	0.15	0.15
**P6(L5/6)**	0.05	0.05	0.15	0.50	1.00
**B6**	0.05	0.05	0.05	0.10	0.50

Table ESM5 Synaptic weights (nS)

Rows: Postsynaptic neurons; columns: Presynaptic neurons

	P2/3	B2/3	NB2/3	SS4(L4)	SS4(L2/3)	P4	B4	NB4	P5(L2/3)	P5(L5/6)	B5	NB5	P6(L4)	P6(L5/6)	B6
**P2/3**	0.020	0.126	0.001	0.356	0.036	0.073	1.080	-	0.004	-	-	-	0.047	-	-
**B2/3**	0.560	0.026	0.001	0.701	0.078	0.161	0.228	-	0.074	-	-	-	0.159	-	-
**NB2/3**	0.408	0.069	0.014	0.872	0.085	0.173	0.581	-	0.159	-	-	-	0.178	-	-
**SS4(L4)**	0.001	0.043	-	0.067	0.092	0.061	0.010	0.003	0.069	0.069	-	-	0.004	-	-
**SS4(L2/3)**	0.001	0.043	-	0.067	0.092	0.061	0.011	0.003	0.069	0.069	-	-	0.004	-	-
**P4**	0.001	0.043	0.008	0.067	0.092	0.061	0.014	0.001	0.069	0.069	-	-	0.004	0.004	-
**B4**	0.101	0.098	-	0.627	0.627	0.627	0.025	0.003	0.841	0.841	-	-	0.062	0.062	-
**NB4**	0.139	0.244	-	0.318	0.318	0.318	0.068	0.013	1.058	1.058	-	-	0.052	-	-
**P5(L2/3)**	0.037	0.188	0.004	0.091	0.082	0.050	0.341	0.005	0.079	0.471	0.459	0.003	0.032	0.093	-
**P5(L5/6)**	0.037	0.188	0.004	0.091	0.082	0.050	0.289	0.005	0.062	0.335	0.416	0.003	0.032	0.093	-
**B5**	0.083	0.098	-	0.274	0.273	0.151	0.191	-	0.910	3.966	0.166	0.003	0.342	0.207	-
**NB5**	0.064	0.244	-	0.331	0.422	0.196	0.521	-	0.603	2.596	0.166	0.014	0.359	0.657	-
**P6(L4)**	0.003	1.045	0.015	0.137	0.145	0.095	0.226	0.001	0.084	0.055	0.293	0.004	0.064	0.062	0.075
**P6(L5/6)**	0.003	1.045	0.015	0.137	0.145	0.095	0.978	0.016	0.201	0.055	0.293	0.004	0.064	0.062	0.048
**B6**	0.123	0.140	-	0.274	0.273	0.151	0.193	-	0.091	0.091	0.021	-	1.105	0.768	0.015

Table ESM6 Synaptic parameters (postsynaptic), from (Traub et al., 2005b)

	E_{AMPA} (mV)	E_{GABA} (mV)	t_{AMPA} (ms)	t_{GABA} (ms)
**P, SS**	0	-75	2.0	6.0
**B, NB**	0	-75	0.8	3.0

Table ESM7 Compartment IDs in each postsynaptic group that presynaptic neurons connect onto (compartment numbers illustrated in Fig. ESM2). Based on (Traub et al., 2005b)

Postsynaptic neurons	Presynaptic neurons: P2/3, P4	SS	P5	P6	B	NB
**P2/3, P4**	3,6,7,8	6-8	4,5	4	1,2,6	3-5,7,8
**SS4**	3,4,6,7	3,4,6,7	3,4,6,7	3,4,6,7	1,2,5	3,4,6,7
**P5**	2-9	2-5	2-5,7-9	2-5,7-9	1,2,7	3-5,7,8
**P6**	2-9	2,4,5	2-9	2-9	1,2,7	3-5,7,8
**B**	3,4,6,7	3,4,6,7	3,4,6,7	3,4,6,7	3,4,6,7	3,4,6,7
**NB**	3,4,6,7	3,4,6,7	3,4,6,7	3,4,6,7	3,4,6,7	3,4,6,7

Table ESM8 Neocortical slice model layer boundaries. Brain surface is at 2600 microns, white matter boundary at 0 microns

Layer	Upper boundary (μm)
1	2600
2/3	2362
4	1835
5	1122
6	832

Table ESM9 Random current input parameters

	Mean current (pA)	Standard deviation (pA)	Noise correlation time constant (ms)
**P2/3**	360	110	2.0
**B2/3**	200	60	0.8
**NB2/3**	160	40	0.8
**SS4**	205	50	2.0
**P4**	250	70	2.0
**B4**	200	60	0.8
**NB4**	160	40	0.8
**P5**	860	260	2.0
**B5**	200	60	0.8
**NB5**	160	40	0.8
**P6**	660	170	2.0
**B6**	200	60	0.8

Table ESM10 List of the extra cell models downloaded from NeuroMorpho.org (Ascoli et al., 2007) used to investigate LFP range and magnitude in Fig. 1 and Fig. 2. Further experimental details are given in the Supplementary Methods, below

Type	NeuroMorpho.Org ID	Species/Region	Original publication
**P2/3**	NMO_00850	Cat visual cortex	Kisvárday and Eysel, 1992
**P2/3**	NMO_00851	Cat visual cortex	Kisvárday and Eysel, 1992
**P2/3**	NMO_00853	Cat visual cortex	Kisvárday and Eysel, 1992
**P2/3**	NMO_00854	Cat visual cortex	Kisvárday and Eysel, 1992
**P2/3**	NMO_00859	Cat visual cortex	Kisvárday and Eysel, 1992
**P2/3**	NMO_00856	Cat visual cortex	Kisvárday and Eysel, 1992
**P2/3**	NMO_00857	Cat visual cortex	Kisvárday and Eysel, 1992
**P2/3**	NMO_00935	Cat visual cortex	Hirsch et al., 2002
**P2/3**	NMO_05813	Cat suprasylvian gyrus	Volgushev et al., 2006
**P2/3**	NMO_05811	Cat suprasylvian gyrus	Volgushev et al., 2006
**P5**	NMO_00880	Cat temporal sulcus	Contreras et al., 1997

Table ESM11 Firing rates in the model under the four conditions illustrated in Fig. 8: model as described, exhibiting gamma oscillation; model with P2/3 -> B2/3 connections at 1% baseline strength; model with B2/3 -> P2/3 connections at 1% baseline strength; model with increased input current to B2/3 neurons (150% mean and standard deviation)

	Baseline	1% P2/3 -> B2/3 weight	1% B2/3 -> P2/3 weight	150% input to B2/3
**P2/3**	3.6	7.0	9.4	0.0
**B2/3**	24.8	1.1	60.5	66.8
**NB2/3**	4.2	8.4	4.0	0.0
**SS4(L4)**	1.3	1.4	1.0	1.0
**SS4(L2/3)**	1.2	1.4	0.7	0.6
**P4**	1.6	1.9	1.0	0.9
**B4**	5.6	9.2	3.4	1.2
**NB4**	2.1	2.6	1.4	0.6
**P5(L2/3)**	3.9	4.3	2.6	2.0
**P5(L5/6)**	3.6	4.3	2.1	1.4
**B5**	16.8	23.4	4.0	0.3
**NB5**	3.3	4.8	0.1	0.0
**P6(L4)**	1.5	2.3	0.7	0.5
**P6(L5/6)**	1.4	1.9	0.7	0.6
**B6**	7.1	9.3	5.7	4.5

SUPPLEMENTARY METHODS

Reduced neuron models

During our investigations with the reduced cell models, we found some inconsistencies when comparing their dynamics with the original cell reconstructions when using the reduced compartment dimensions given in (Bush and Sejnowski, 1993). We therefore recalculated the compartment lengths and diameters from the three cell types specified in (Mainen and Sejnowski, 1996) using the method specified in (Bush and Sejnowski, 1993). For these calculations, we used a version of the NEURON code originally written by Alain Destexhe to reduce a compartmental model to 3 compartments (Destexhe et al., 1998), modified by Michael Hines to work for any number of compartments. This code implements the method described in (Bush and Sejnowski, 1993). Michael Hines' version of this code is available on the NEURON forum at http://www.neuron.yale.edu/phpbb/viewtopic.php?f=13&t=589. The recalculated cell dimensions are given in Table ESM1. We used our recalculated dimensions both for the LFP simulation comparison between the cell reconstructions and reduced cell models and for the necortical slice model.

Comparison of LFPs from reduced & full neuron morphologies

Fig. 1 and Fig. 2 show comparisons between LFPs calculated using populations containing reduced and full neuron models, and include further comparisons with extra (full-scale) neurons to place the results in the context of general biological variability (experiments described in the main text). These extra neurons were downloaded from NeuroMorpho.Org (Ascoli et al., 2007) and are listed in Table ESM10, above. Prior to being simulated in LFPy, we first removed all axonal compartments and rotated the neurons so that their apical dendrites were approximately parallel with the *z*-axis.

Connectivity

The connectivity model for the neocortical slice model (implemented in VERTEX) is described in the main text; here we give details of how neurons spanning multiple layers are connected. Pyramidal neuron dendrites span several layers above their soma layer, and connectivity statistics are provided per layer for pyramidal cells in (Binzegger et al., 2004) – see Table ESM3. As all neurons within a population are the same size, but have different soma positions, each neuron's compartments will cross the model's layer boundaries at different points. For simplicity, we ignore this variability for the purposes of connecting up the model, defining the layers in which a compartment resides based on its position when its neuron's soma is positioned in the centre of its layer. If several compartments could be chosen, then the compartment on which the synapse is made is chosen randomly, with each possible compartment having a probability of being selected equal to the membrane area of the compartment in the layer divided by the neuron's total membrane area in the layer. The chosen compartment must also be allowed according to Table ESM7. For our simulations, we allowed multiple synapses between a single pre- and postsynaptic neuron pair (targets randomly chosen with replacement), but did not allow autapses. These options can be configured in the simulation parameters.

Neuron and synapse dynamics

For the dynamics simulation of the neocortical slice model, we chose the 2 variable adaptive exponential (AdEx) model (Brette and Gerstner, 2005). The AdEx model can reproduce most of the dynamical features exhibited by cortical neurons (Naud et al., 2008), all its parameters have a direct biological correlate (Gerstner and Brette, 2009) making the model easy to interpret and modify in light of new experimental data, its sub-threshold behaviour is realistic (Badel et al., 2008), and its bifurcation structure is well characterised and is the same as the commonly used Izhikevich model (Naud et al., 2008; Touboul and Brette, 2008). It can be extended to include passive dendrite compartments (Clopath et al., 2007; Gerstner and Brette, 2009) required for LFP simulation, which we did by incorporating the AdEx dynamics into the somatic compartment of the passive cell model reductions described above. A modification of the AdEx model was also used recently in another study of gamma oscillations (Economo and White, 2012).

Each neuron is modelled as a passive cable structure of cylindrical compartments, with the AdEx spiking mechanism at the soma compartment. Passive parameters are given above. The somatic membrane potential v_s evolves according to equation ESM1:

$$C_s \frac{dv_s}{dt} = -g_{leak,s}\left(v_s - E_{leak}\right) - \sum_j g_{sj}\left(v_s - v_j\right) + g_{leak,s}\Delta_t \exp\left(\frac{v_s - V_t}{\Delta_t}\right) - w + I_s, \qquad \textbf{(ESM1)}$$

$$\tau_w \frac{dw}{dt} = \alpha\left(v_s - E_{leak}\right) - w,$$

if $v_s \geq v_{cutoff}$:

$v_s \leftarrow v_{reset}$,

$w \leftarrow w + \beta$,

where C_s is the soma membrane capacitance, $g_{leak,s}$ is the soma leak conductance (= reciprocal of soma membrane resistance), E_{leak} is the leak reversal potential, g_{sj} are the conductances between the soma and its j connected compartments, v_j is the membrane potential of the jth connected compartment, Δ_t is a constant defining the spike steepness, V_t is the instantaneous threshold potential, w is a current representing the combined slow ionic currents, I_s is the total current input at the soma (from synaptic and externally applied currents), τ_w is the time constant of the slow current w, α is the scale factor of the slow current, v_{cutoff} is the potential at which a spike is said to have been fired, v_{reset} is the membrane potential to which v_s returns after a spike, and β is the instantaneous change in the value of the slow current w after a spike (Brette and Gerstner, 2005). All dendrites are passive:

$$C_k \frac{dv_k}{dt} = -g_{leak,k}\left(v_k - E_{leak}\right) - \sum_j g_{kj}\left(v_k - v_j\right) + I_k, \qquad \textbf{(ESM2)}$$

where the symbols are as before, for dendritic compartment k rather than soma s.

We adjusted the parameters for each neuron type to produce similar spiking patterns to the model neurons described in (Traub et al., 2005b). Each cell type's passive parameters were defined by its morphology and the electrotonic parameters given in Table ESM2; therefore, the parameters adjusted to fit the spiking responses of the Traub neurons were the spike slope factor Δ_t, threshold V_t, adaptation time constant τ_w, adaptation coupling parameter α, reset value v_{reset}, and instantaneous adaptation current increase β. We employed a qualitative approach to parameter adjustment, guided by the analysis of the AdEx model in (Naud 2008). According to the classifications in (Naud 2008), B, SS and P6 cells have a sharp reset, while NB, P2/3, P4 and P5 cells have a broad reset. B cells are non-adapting; SS and P6 cells are adapting; P2/3, P4, P5 and NB cells show an initial burst. These properties were chosen based on the membrane potential traces reported in Appendix A of (Traub et al., 2005b).

The model includes AMPA and $GABA_A$ synapses, each modelled as single exponential, conductance-based synapses. When a neuron fires a spike, the synaptic conductances (g_{AMPA} for excitatory presynaptic neurons, g_{GABA} for inhibitory presynaptic neurons) at the contacted target compartments k (specified in the connectivity matrix) are increased by the synaptic weight (specified in the weights matrix) after the relevant axonal delay time, then decay exponentially:

$$\frac{dg_{AMPA,k}}{dt} = -\frac{g_{AMPA,k}}{\tau_{AMPA}}, \qquad \textbf{(ESM3)}$$

$$\frac{dg_{GABA,k}}{dt} = -\frac{g_{GABA,k}}{\tau_{GABA}},$$

where τ_{AMPA} and τ_{GABA} are the AMPA and $GABA_A$ synaptic decay constants, respectively (specified in Table ESM6). As we assume that the conductances of individual synapses sum linearly in each compartment, we only need one variable per type of synaptic conductance per compartment, rather than keeping track of all synapses individually. The total synaptic current I_k at compartment k at time t is then given by

$$I_k(t) = g_{AMPA,k}\left(v_k(t) - E_{AMPA}\right) + g_{GABA,k}\left(v_k(t) - E_{GABA}\right), \qquad \textbf{(ESM4)}$$

where E_{AMPA} and E_{GABA} are the reversal potentials for AMPA and $GABA_A$, respectively. Synaptic weights (Table ESM5) were chosen based on those reported in (Traub et al., 2005b), scaling the weights according to the number of synapses between groups in our model compared with the Traub model. Our neuron populations did not match theirs exactly, with the following differences (in addition to different numbers of neurons and

synapses): our model includes interneurons in every layer, while the Traub model has only "superficial" and "deep" interneurons (with the deep interneurons providing inhibition to layer 4); the Traub model only has spiny stellate cells in layer 4 (no pyramidal or interneurons); the Traub model contains fast rhythmic bursting pyramidal cells in layer 2/3 and intrinsically bursting pyramidal cells in layer 5 – our model contains no bursting neurons; our model contains synapses between some neuron groups that are not present in the Traub model. We therefore had to make several arbitrary decisions when setting some synapse weights between groups.

Model input

We stimulate our model to mimic the bath application of kainate. This stimulates the pyramidal cell axonal plexus, providing the neurons with excitatory drive. We simulate this by applying independent random input currents I_{Ki} to each neuron i, modelled as Ornstein-Uhlenbeck processes similar to (Arsiero et al., 2007) using Gillespie's exact discretisation method:

$$I_{Ki}(t+\delta t) = I_{Ki}(t) + \left(1-\exp\left(\frac{-\delta t}{\tau_K}\right)\right)\times\left(m_{Ki} - I_{Ki}(t)\right) + \sqrt{1-\exp\left(-2\frac{\delta t}{\tau_K}\right)} \times S_{Ki} \times N_{Ki}(0,1), \qquad \textbf{(ESM5)}$$

(Gillespie, 1996), where δt is the length of the time step, τ_k is the noise correlation time constant, m_{Ki} is the mean current value, S_{Ki} is the standard deviation and $N_{Ki}(0,1)$ is a normally distributed random number. The random current is distributed across the neuron's compartments proportionally to the compartment membrane areas. Any currents falling below zero are reset to zero for that time step, so that the input current is always either positive or zero.

LFPs are calculated by summing the membrane currents of each compartment, weighted by distance from the electrode tips (described in the main text). The membrane current $I_{mem,k}$ of compartment k is just the negative of the axial current $I_{ax,k}$ entering the compartment (Johnston and Wu, 1995):

$$I_{mem,k} = \sum_j g_{kj}\left(v_k - v_j\right) = -I_{ax,k}. \qquad \textbf{(ESM6)}$$

Model implementation

The neocortical slice model was implemented in our VERTEX simulation tool. VERTEX is written in Matlab, using the Matlab Parallel Computing Toolbox for parallelisation, though it can also be run serially. It is designed to be easy to use: neuron groups, connectivity patterns, model size/layers and simulation settings are defined in Matlab structures, requiring minimal programming ability to specify models of different cortical areas, explore the parameter space, or apply different stimuli.

First, the model is initialised by distributing neurons across parallel processes, positioning the neurons, setting up the connectivity matrix, and calculating axonal delays. Next, the electrode locations are specified and distances between each electrode and each compartment are calculated, using either the point distance for somas, or the line source distance for dendrites. Pre-calculating the constant values used in the field potential calculations minimises the impact of calculating the LFP during the simulation.

We used the methods outlined in (Morrison et al., 2005) for parallel simulation. These minimise communication overhead by storing synapse information (delays, postsynaptic neuron IDs and compartment IDs) on the postsynaptic side, so only spiking presynaptic IDs and timestamps need to be exchanged between processes. Spikes do not need to be delivered every time step: assuming the minimum synaptic delay is $d_{\min} \cdot h$ (where h is the step size), spikes can be buffered on the pre-synaptic side and exchanged every $d_{\min}$ time steps. Processes communicate using the complete pairwise exchange algorithm (Tam & Wang, 2000; Morrison et al., 2005).

The simulation made use of vectorised data structures and algorithms in order to keep run-times reasonable (Brette and Goodman, 2011). The benefits of vectorisation increase with model size as fewer interpretation overheads are incurred per variable. In addition to the methods described in (Brette and Goodman, 2011), which do not include compartmental neuron models, we vectorise the calculation of the axial currents between compartments.

SUPPLEMENTARY REFERENCES

Badel L, Lefort S, Brette R, Petersen CC, Gerstner W, Richardson MJ (2008). Dynamic I-V curves are reliable predictors of naturalistic pyramidal-neuron voltage traces. J Neurophysiol 99 (2):656-666

Clopath C, Jolivet R, Rauch A, Luscher HR, Gerstner W (2007). Predicting neuronal activity with simple models of the threshold type: Adaptive exponential integrate-and-fire model with two compartments. Neurocomputing 70:1668-1673

Contreras D, Destexhe A, and Steriade M (1997). Intracellular and computational characterization of the intracortical inhibitory control of synchronized thalamic inputs *in vivo*. J Neurophysiol 78 (1):335-350

Destexhe A, Neubig M, Ulrich D, Huguenard J (1998). Dendritic low-threshold calcium currents in thalamic relay cells. J Neurosci 18 (10):3574-3588

Economo MN, White JA (2012). Membrane properties and the balance between excitation and inhibition control gamma-frequency oscillations arising from feedback inhibition. PLOS Comput Biol 8 (1):e1002354

Gerstner W, Brette R (2009). Adaptive exponential integrate-and-fire model. Scholarpedia 4:8427

Gillespie DT (1996). Exact numerical simulation of the Ornstein-Uhlenbeck process and its integral. Phys Rev E 54 (2):2084-2091

Hirsch JA, Martinez LM, Alonso JM, Desai K, Pillai C, Pierre C (2002). Synaptic physiology of the flow of information in the cat's visual cortex *in vivo*. J Physiol 540 (Pt 1):335-350

Kisvárday ZF, Eysel UT (1992). Cellular organization of reciprocal patchy networks in layer III of cat visual cortex (area 17). Neuroscience 46 (2):275-286

Naud R, Marcille N, Clopath C, Gerstner W (2008). Firing patterns in the adaptive exponential integrate-and-fire model. Biol Cybern 99:335-347

Tam ATC, Wang C-L (2000). Efficient scheduling of complete exchange on clusters. In: Chaudry G, Sha E (eds) 13th International Conference on Parallel and Distributed Computing Systems (PDCS 2000), International Society for Computers and Their Applications, Las Vegas, Nevada, pp 111-116

Touboul J, Brette R (2008). Dynamics and bifurcations of the adaptive exponential integrate-and-fire model. Biol Cybern 99:319-334

Volgushev M, Chauvette S, Mukovski M, Timofeev I (2006). Precise long-range synchronization of activity and silence in neocortical neurons during slow-wave oscillations. J Neurosci 26 (21):5665-5672
